# From economic burden to patient-centered solutions: the evolving landscape of financial toxicity in oncology

**DOI:** 10.3389/fpubh.2026.1806145

**Published:** 2026-06-08

**Authors:** Luya Shi, Yu Zhang, Chengdong Sun, Jianying Dai

**Affiliations:** 1 Municipal Hospital Affiliated to Taizhou University, Taizhou Key Laboratory of Infection and Tumor Immunology, Taizhou, Zhejiang, China; 2 Xinjiang Clinical Research Center for Perinatal Diseases, Urumqi Maternal and Child Health Hospital, Urumqi, Xinjiang, China; 3 The Second Affiliated Hospital of Zhejiang Chinese Medical University, Hangzhou, Zhejiang, China

**Keywords:** bibliometric analysis, cancer, financial burden, financial toxicity, tumor

## Abstract

**Background:**

Financial burden has emerged as a significant challenge in oncology, adversely affecting patients’ quality of life, treatment adherence, and long-term outcomes. While research on cancer-related financial toxicity (FT) has grown rapidly, a comprehensive bibliometric analysis of its knowledge structure, thematic evolution, and emerging trends remains scarce.

**Methods:**

Publications on FT among cancer patients, published from 2003 to January 19, 2026, were retrieved from the Web of Science Core Collection and Scopus databases. Bibliometric and science mapping analyses were conducted using CiteSpace, VOSviewer, and R software to assess publication trends, collaboration networks, research hotspots, and thematic evolution.

**Results:**

Research on FT in cancer patients has experienced substantial growth over the past two decades, with increasing interdisciplinary integration. The focus has shifted from descriptive economic burden assessments to patient-centered and system-oriented approaches, conceptualizing FT as a modifiable risk embedded within social and clinical contexts. Social support and multidisciplinary management have emerged as major interconnected research hotspots, emphasizing that effective mitigation of FT requires addressing both upstream social determinants of health and downstream integration of coordinated clinical interventions. Emerging keywords suggest that measurement and conceptualization, behavioral consequences, socioeconomic impact, and intervention & health system response are likely to influence future research directions.

**Conclusion:**

This bibliometric analysis reveals that FT research has evolved into a comprehensive conceptual framework, positioning FT as a modifiable, system-level risk in cancer care. By synthesizing evidence on social support and multidisciplinary management, this study proposes a model linking social determinants, clinical assessment, and coordinated interventions. These findings offer valuable insights to guide future research and the development of targeted strategies aimed at alleviating financial burden and improving survivorship care, supporting the integration of FT management into patient-centered oncology practice.

## 1 Introduction

Cancer represents a major global public health challenge, profoundly impacting population health and quality of life worldwide. Advances in early detection, coupled with the rapid development of targeted therapies and immunotherapies, have significantly improved survival rates, leading to growing recognition of cancer as a condition necessitating long-term, chronic disease management (1, 2). As of January 1, 2025, an estimated 18.6 million individuals in the United States are living with a history of cancer, with projections indicating that this number will exceed 22 million by 2035 (3). These survival improvements, however, have been accompanied by a persistent increase in cancer-related healthcare expenditures, with total cancer care costs in the United States expected to reach approximately USD 246 billion by 2030 (4, 5). The rising financial demands associated with cancer diagnosis and treatment have placed significant economic strain on patients and their families, resulting in poorer quality of life, reduced treatment adherence, care delays or discontinuation of care, psychological distress, and higher mortality risk. These financial burdens are now recognized as critical non-biomedical determinants of cancer outcomes (6, 7). Therefore, the financial burden experienced by cancer patients is not only prognostic but also intricately linked to the delivery of high-quality oncology care, encompassing the affordability of services, antineoplastic therapies, and palliative care (8). Addressing cancer-related financial burdens has thus become a cornerstone of quality cancer care and a key element in achieving the United Nations’ goal of universal health coverage (UHC), with profound implications for population health and health equity.

Within this framework, financial toxicity (FT) has emerged as a key construct to capture the financial distress faced by cancer patients and their families and its detrimental impact on health outcomes (9). FT encompasses a wide range of economic consequences, including out-of-pocket medical expenses, indirect costs, financial coping strategies, asset depletion, and changes in household financial stability due to cancer (9). FT spans the entire cancer care continuum, from diagnosis and active treatment to supportive care, palliative care, and survivorship (8). Globally, approximately half of all cancer patients experience some degree of FT, affecting 30–50% of patients in high-income countries and 70–80% in low- and middle-income countries, with socioeconomically disadvantaged populations bearing a disproportionate burden (10). Key factors associated with FT include income level, insurance coverage, age, education, cancer type, treatment modality, and regional economic context (11). Although definitions and measurement approaches vary across settings and studies, both subjective and objective indicators are commonly employed to assess FT. Widely used instruments include the Comprehensive Score for Financial Toxicity (COST), the Patient-Reported Outcome for Fighting Financial Toxicity (PROFFIT), and the Subjective Financial Distress Questionnaire (SFDQ) (12, 13). While the mechanisms linking FT to adverse survival outcomes are not fully understood, they are believed to involve reduced treatment adherence, diminished continuity of follow-up care, and an increased risk of adverse events due to compromised care management capacity (14). In recent years, there has been growing emphasis on intervention strategies and policy responses aimed at mitigating FT (15, 16). Interventions such as financial navigation programs, integration of social work services, insurance expansion, drug price negotiations, reduction of low-value care, and patient education have shown potential in improving patient experience and treatment adherence; however, robust evidence evaluating their effectiveness remains limited (17). At the policy level, initiatives such as public insurance schemes, patient assistance programs, and increased cost transparency have been introduced in some countries, though their overall impact has been inconsistent (18). Alongside increased clinical and policy awareness, research on FT in cancer care has expanded rapidly, covering a range of dimensions including epidemiological assessments, analyses of contributing factors, development and validation of measurement tools, evaluation of health outcomes, and exploration of intervention strategies (19, 20). Despite this expansion, there has been no comprehensive synthesis or visual mapping of the global research landscape, knowledge structure, thematic evolution, and patterns of interdisciplinary collaboration within this field. A systematic examination of the development and emerging trends in FT research is therefore essential to support precision-oriented nursing interventions and promote health equity.

Bibliometric analysis, grounded in quantitative methods and scientific knowledge mapping, offers a robust framework for characterizing the evolution of a research field by examining publication output, collaboration networks among authors and institutions, core journals, highly cited references, and keyword co-occurrence patterns (21). In addition to identifying influential studies and research hotspots, bibliometric approaches can shed light on thematic trajectories and emerging research frontiers (21). While bibliometric methods have been successfully applied across various medical research domains, existing bibliometric and mapping studies on financial toxicity have primarily focused on specific cancer types or narrower research contexts, such as breast cancer (22, 23). A comprehensive cross-cancer bibliometric analysis integrating the broader FT literature across oncology remains lacking. Therefore, this study integrates bibliometric techniques with knowledge visualization methods, synthesizing literature from two major international databases, the Web of Science Core Collection (WoSCC) and Scopus. The study aims to identify key contributors and influential publications, systematically map the global research landscape and developmental trends of FT in cancer care, and highlight emerging hotspots and frontiers. By providing a comprehensive overview of this rapidly evolving field, this study seeks to inform future research priorities, guide resource allocation and policy decision-making, and ultimately support evidence-based strategies to alleviate the financial burden of cancer and improve patient quality of life.

## 2 Materials and methods

### 2.1 Data collection

**Figure 1:**
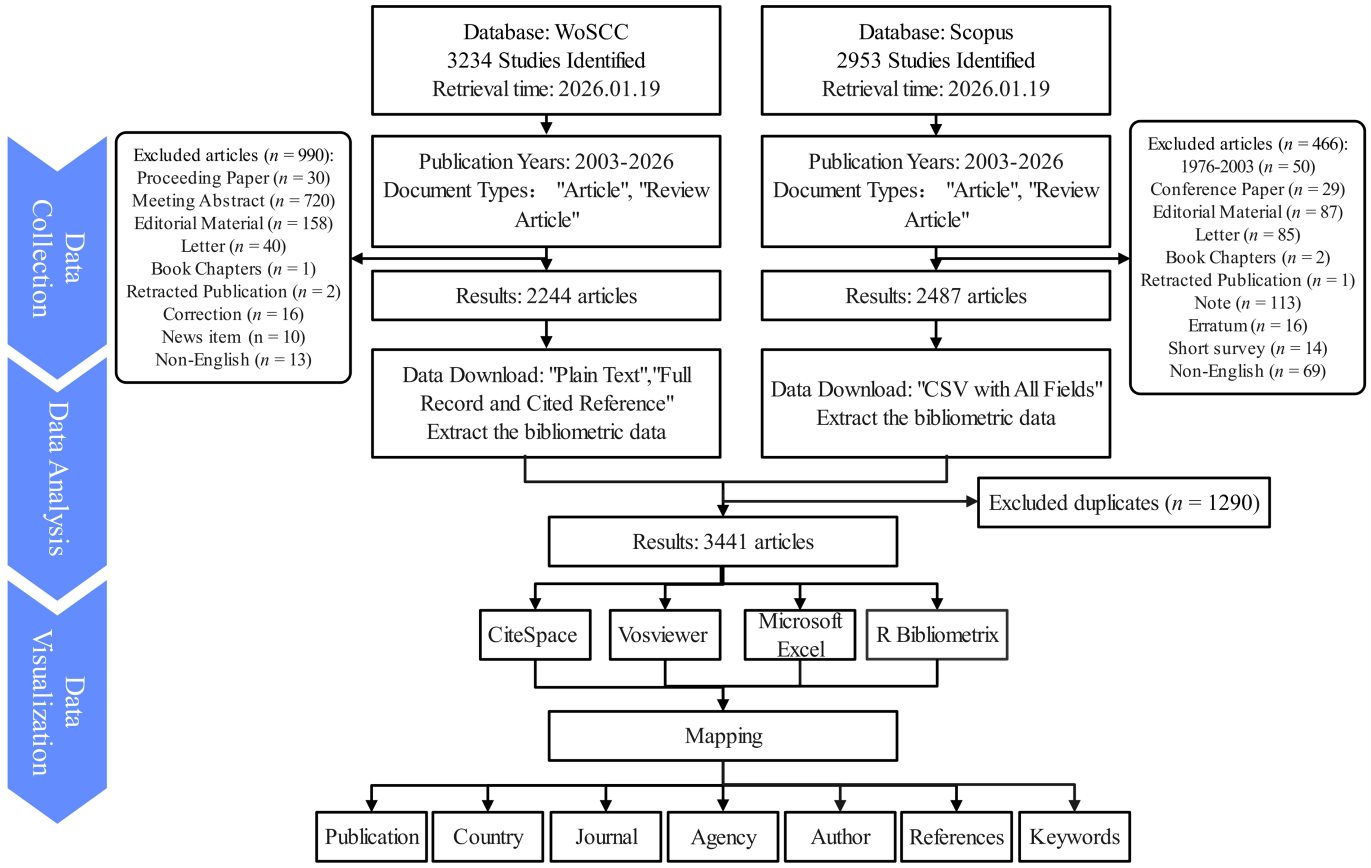
Study selection process for literature on financial toxicity in cancer patients.

The WoSCC and Scopus databases are among the most authoritative and representative sources of high-impact scholarly literature worldwide, commonly utilized in bibliometric research due to their extensive coverage and data reliability. In this study, publications on FT among cancer patients were retrieved from both databases for bibliometric analysis. FT is generally defined as the financial burden and financial distress experienced by patients and their families as a result of cancer diagnosis and treatment. To ensure the comprehensiveness and precision of the search strategy, keywords were initially identified through an extensive literature review and refined by incorporating relevant Medical Subject Headings (MeSH) terms from PubMed. The selection of search terms was guided by the established conceptual framework of FT, which encompasses objective financial burden, indirect economic consequences, and subjective financial distress experienced by patients. Accordingly, in addition to the core term “financial toxicity,” a range of related terms reflecting patient-level financial burden and distress were included, such as “financial burden,” “financial stress,” “financial hardship,” and “economic hardship,” to capture the broader conceptual domain of FT. Synonyms and variations of these terms were combined using Boolean operators to maximize sensitivity and ensure comprehensive retrieval of relevant literature. It should be noted that this study did not directly involve input from cancer survivors in the selection of search terms. However, the search strategy was informed by prior studies that incorporated patient-reported outcomes (PROs) and qualitative assessments of financial distress, thereby indirectly reflecting the patient perspective embedded in the FT literature. Exploratory searches in WoSCC revealed that research in this field began to emerge around 2003, prompting the time frame for literature retrieval to be set from 2003 onward. On January 19, 2026, a comprehensive search was performed in the WoSCC database using the following search strategy: TS = (“Financial Stress*” OR “Economic Burden*” OR “Financial Burden*” OR “Financial Toxicit*” OR “Financial Challenge*” OR “Financial Pressure*” OR “Financial Strain*” OR “Insufficient Financial Resource*” OR “Financial Resource Strain*” OR “Socioeconomic Adversit*” OR “Financial Hardship*” OR “Economic Hardship*”) AND TS = (“Tumor*” OR “Neoplasia*” OR “Neoplasm*” OR “Cancer*” OR “Malignant Neoplasm*” OR “Malignanc*”). This search yielded a total of 3,234 records. Filters were then applied to include only articles and reviews, while excluding retracted publications and book chapters. The publication years were limited to 2003–2026, resulting in 2,244 eligible publications for further analysis (Figure 1). A parallel search was conducted in the Scopus database using the following search strategy: TITLE-ABS-KEY (“Tumor*” OR “Neoplasia*” OR “Neoplasm*” OR “Cancer*” OR “Malignant Neoplasm*” OR “Malignanc*”) AND TITLE-ABS-KEY (“Financial Stress*” OR “Economic Burden*” OR “Financial Burden*” OR “Financial Toxicit*” OR “Financial Challenge*” OR “Financial Pressure*” OR “Financial Strain*” OR “Insufficient Financial Resource*” OR “Financial Resource Strain*” OR “Socioeconomic Adversit*” OR “Financial Hardship*” OR “Economic Hardship*”). Database-specific adaptations, including field tags, wildcard handling, and indexing structures, were adjusted according to the requirements of each database to ensure search consistency and comparability. After applying identical inclusion and exclusion criteria, a total of 2,487 publications were retrieved from Scopus.

The WoSCC search results were exported in Plain Text File format, containing Full Records and Cited References, while the Scopus records were exported in “CSV with All Fields.” Records from both databases were combined and managed using EndNote 2025 based on Digital Object Identifiers (DOIs), article titles, and author(s). Following automated deduplication, all potentially duplicated records were manually reviewed by two independent researchers to identify residual duplicates and minimize false duplicate removal caused by minor inconsistencies in metadata formatting. Prior to bibliometric analysis, additional data cleaning and standardization procedures were performed to improve the reliability of network analyses. Synonymous keywords and spelling variations were unified using thesaurus-based normalization where applicable, and inconsistencies in author names and institutional affiliations were manually standardized when necessary. The final dataset consisted of 3,441 unique publications for further analysis. To ensure data reliability, two researchers independently conducted the literature search, document download, and bibliometric data extraction on the same day. Any discrepancies were resolved through consensus discussions with a third researcher.

### 2.2 Data analysis

Bibliometric and visualization analyses were conducted using VOSviewer (version 1.6.20), CiteSpace (version 6.4. R1), the Bibliometrix package, R software, and Microsoft Excel 2019. These tools were used in a complementary manner to ensure comprehensive and reproducible analysis. Specifically, VOSviewer was applied for network construction and visualization, CiteSpace for temporal and structural analysis, and R and Excel for statistical analysis and data preprocessing. The combined use of VOSviewer and CiteSpace enabled the integration of network structure analysis and temporal trend detection, thereby enhancing the robustness and interpretability of the bibliometric results.

#### 2.2.1 CiteSpace

CiteSpace, a Java-based bibliometric and visualization software, is extensively used to explore the structural and temporal characteristics of scientific knowledge (24–26). In this study, CiteSpace was employed to analyze various dimensions, including countries, institutions, authors, journals, references, and keywords. Structural indicators, such as betweenness centrality, were analyzed, while temporal indicators included citation bursts, timeline views, landscape views, and timezone visualizations. Additionally, cluster evolution and dependency analyses were conducted to examine the developmental trajectories of research themes. In the visualized networks, node size represents the frequency of occurrence, and the thickness of links indicates the strength of relationships between nodes. The main parameter settings were as follows: Years Per Slice was set to 1, with selection criteria defined using the g-index and *k* = 25. Default parameters were used for all other settings unless otherwise specified.

#### 2.2.2 VOSviewer

VOSviewer, a widely adopted tool for bibliometric analysis and network visualization (27), was primarily used in this study to construct author collaboration networks and keyword co-occurrence networks. Compared with CiteSpace, VOSviewer is particularly advantageous in handling large-scale data and generating clear and intuitive network visualizations. Relationships among items were visualized using nodes and links of varying sizes and colors, where node size reflects the frequency of occurrence and link thickness represents the strength of associations between items.

## 3 Results

### 3.1 Annual publication output and growth trends

**Figure 2:**
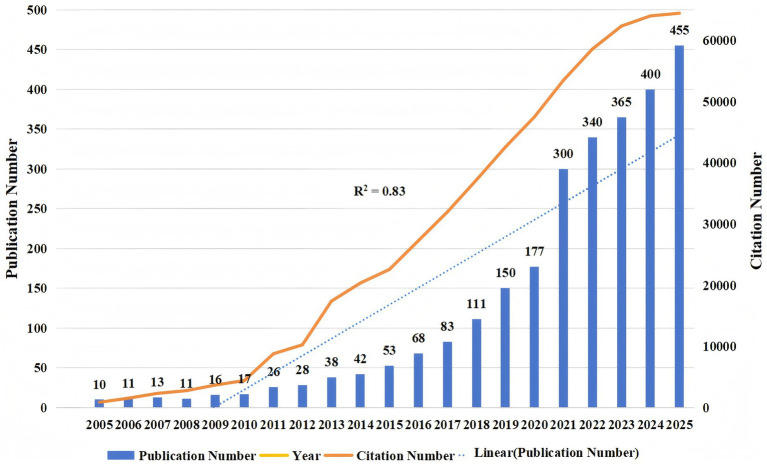
Trends and projected growth of research on financial toxicity in cancer patients. Annual publications and citations related to financial toxicity in cancer patient from 2003 to 2026. The bar chart represents the annual number of publications, while the solid line represents the yearly citation number.

Analysis of annual publication output and temporal trends provides valuable insights into the academic significance and global research activity in a particular field (25). Between 2003 and 2026, a total of 3,441 publications on FT among cancer patients were published across 978 journals, authored by 15,159 researchers. The average document age was 5.18 years, and the average number of citations per document was 24.03, reflecting the rapid expansion of research in this area and its significant scholarly impact. Over this period, the annual number of publications demonstrated a clear linear growth trend, with a coefficient of determination (*R*^2^ = 0.83) and an annual growth rate of 9.64% (Figure 2). The number of publications increased from 4 articles in 2003 to 563 articles in 2025, marking an approximately 140-fold rise. Notably, the growth rate accelerated sharply after 2021, indicating a phase of intensified research activity. Citation counts also followed a steady upward trajectory, with notable peaks corresponding to the publication of highly cited articles in 2011 and 2013.

### 3.2 Analysis of countries and institutions

**Figure 3:**
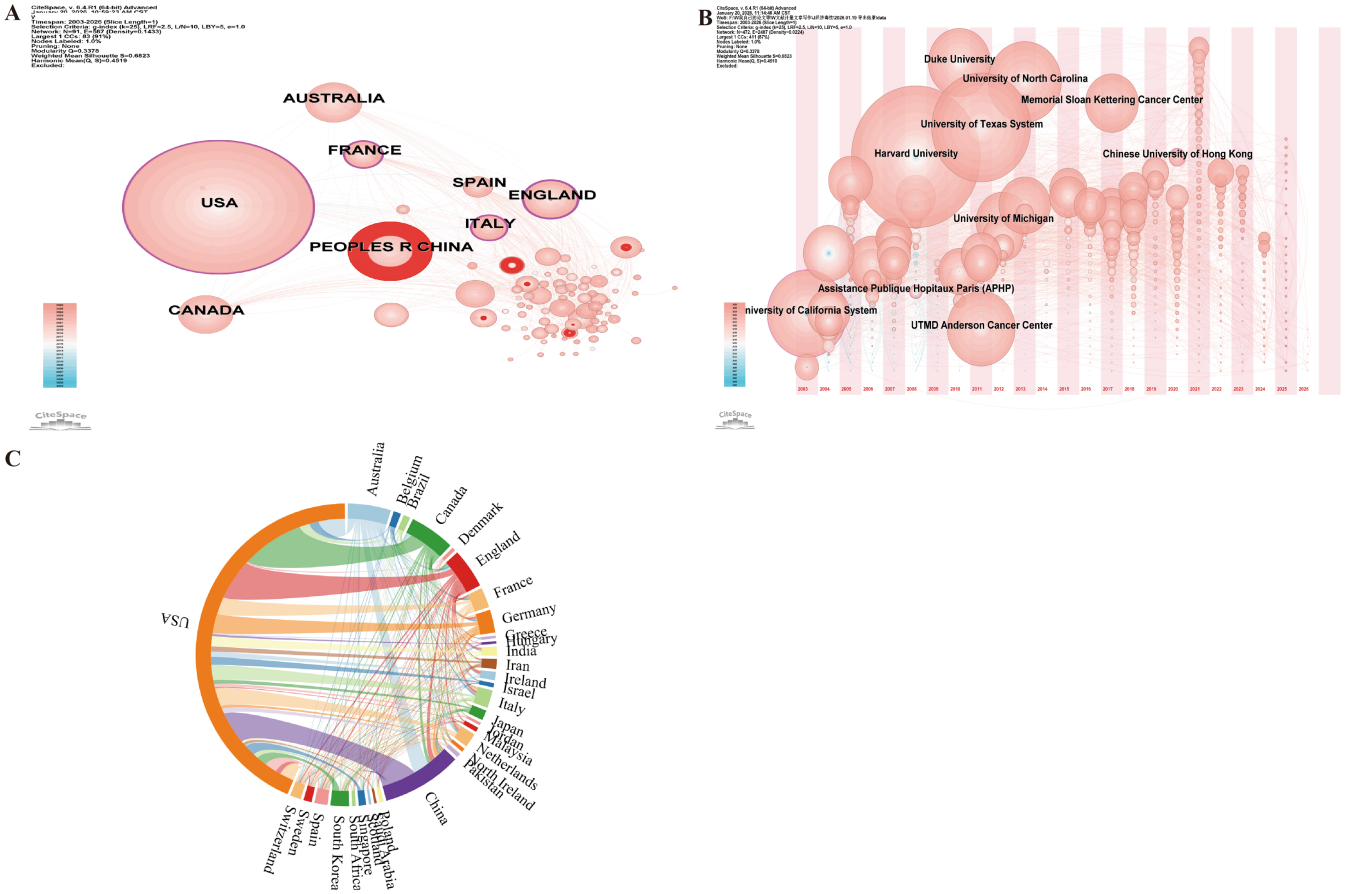
Global distribution and collaboration patterns of research on financial toxicity in cancer patients. **(A)** Distribution of contributing countries. Node size corresponds to the frequency of co-occurrence, while nodes encircled in purple indicate high centrality (≥ 0.1), signifying influential bridging roles. **(B)** Institutional research timeline. The horizontal axis represents the year of the institution’s first published article, with node size indicating the number of articles published. Blue nodes signify earlier publications, while pink nodes indicate more recent ones. Overlapping colors reflect multiple publications within a given year, with a higher count forming rings, symbolizing continuous and extensive publication activity over time. **(C)** International collaboration network, where link thickness indicates the strength of collaboration.

Country and institutional analyses offer insight into the most influential contributors in the field of FT among cancer patients, as well as their collaborative relationships. Publications in this field originated from 91 countries. Seven countries stood out with relatively high betweenness centrality, indicating their pivotal roles in global collaboration networks (Figure 3A): England (0.24), USA (0.19), France (0.15), Canada (0.10), Australia (0.10), Italy (0.10), and Spain (0.10). At the institutional level, key organizations such as Assistance Publique Hôpitaux Paris (APHP) (0.14), University of California System (0.11), Chinese University of Hong Kong (0.11), and Duke University (0.10) played central roles in connecting collaborative research efforts (Figure 3B). In terms of publication output, the top ten most productive institutions were: Harvard University (172), University of Texas System (132), University of California System (105), University of North Carolina (98), Harvard Medical School (93), UTMD Anderson Cancer Center (91), Duke University (83), University of Michigan (72), Memorial Sloan Kettering Cancer Center (71), and University System of Ohio (69). The institutional timezone visualization revealed that the University of California System was among the earliest institutions to publish research on FT in cancer patients.

A collaboration analysis was conducted among the top 30 countries, each with more than nine publications (Figure 3C). The results showed strong collaborative ties between the United States and Canada, the United Kingdom, and China. However, the proportion of international co-authorship was relatively low (12.38%), indicating that cross-national collaboration in this field is still limited and should be further strengthened.

### 3.3 Analysis of authors

**Figure 4:**
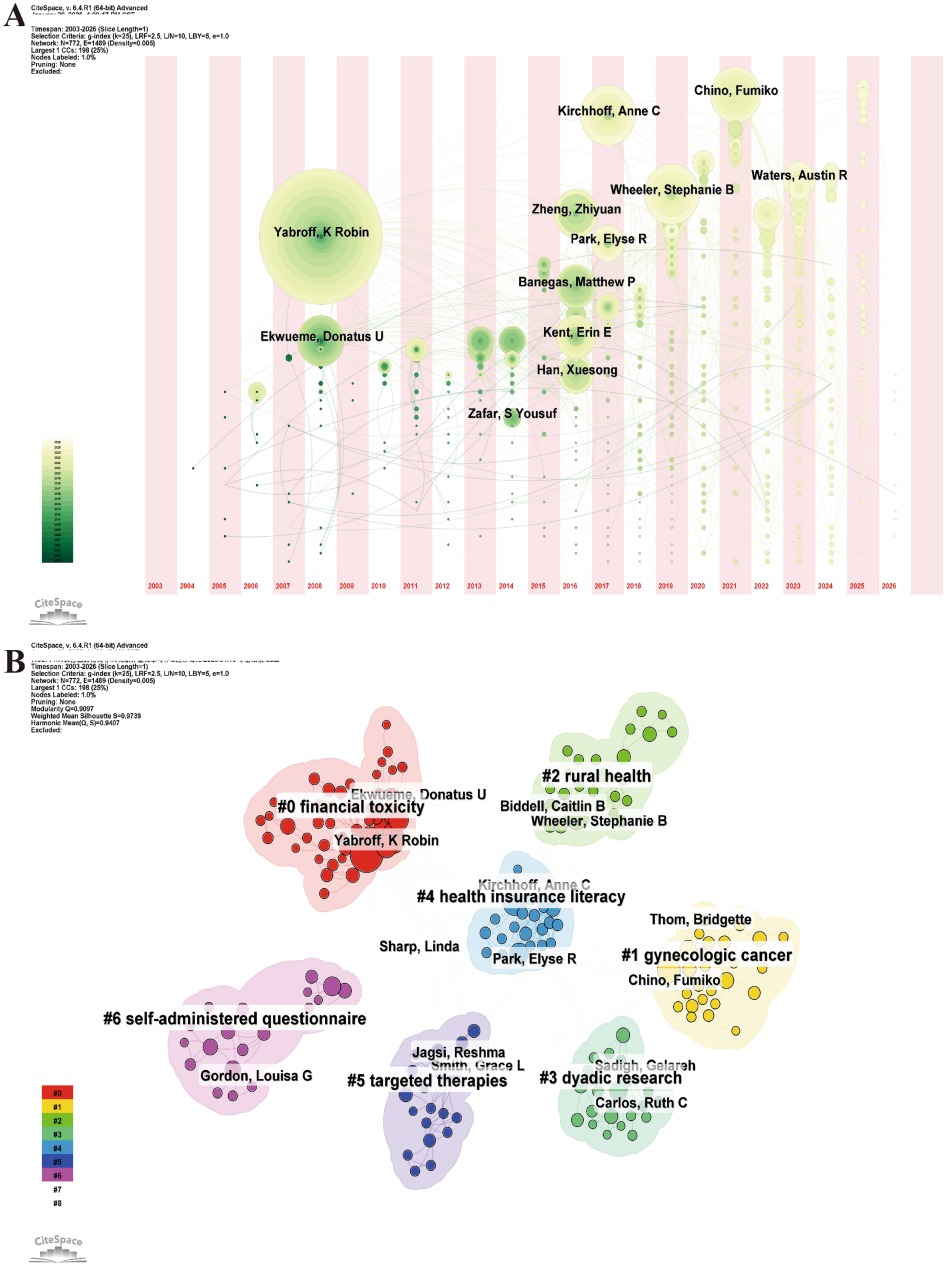
Author collaboration patterns and research clusters in research on financial toxicity in cancer patient. **(A)** Author collaboration over time. **(B)** Author research clusters formed based on author collaboration and the similarity of their research.

Author analysis identifies influential experts and core research teams within the field, facilitating the tracking of research trends and promoting academic collaboration. The ten most productive authors were Yabroff, K. Robin (56), Kirchhoff, Anne C (25), Wheeler, Stephanie B (25), Chino, Fumiko (23), Ekwueme, Donatus U (21), Zheng, Zhiyuan (20), Banegas, Matthew P (18), Kent, Erin E (18), Han, Xuesong (16), and Park, Elyse R (15) (Figure 4A).

Author clustering analysis, based on research themes and collaboration patterns, identified seven distinct clusters (Figure 4B). The largest cluster, “financial toxicity,” was led by Yabroff, K. Robin and Ekwueme, Donatus U. The second-largest cluster, “gynecologic cancer,” was primarily represented by Chino, Fumiko and Thom, Bridgette. The third cluster, “rural health,” centered around Biddell, Caitlin B. and Wheeler, Stephanie B. The fourth cluster, “dyadic research,” was dominated by Sadigh, Gelareh and Carlos, Ruth C. The fifth cluster, “health insurance literacy,” was led by Kirchhoff, Anne C. and Park, Elyse R.

### 3.4 Analysis of journals

**Figure 5:**
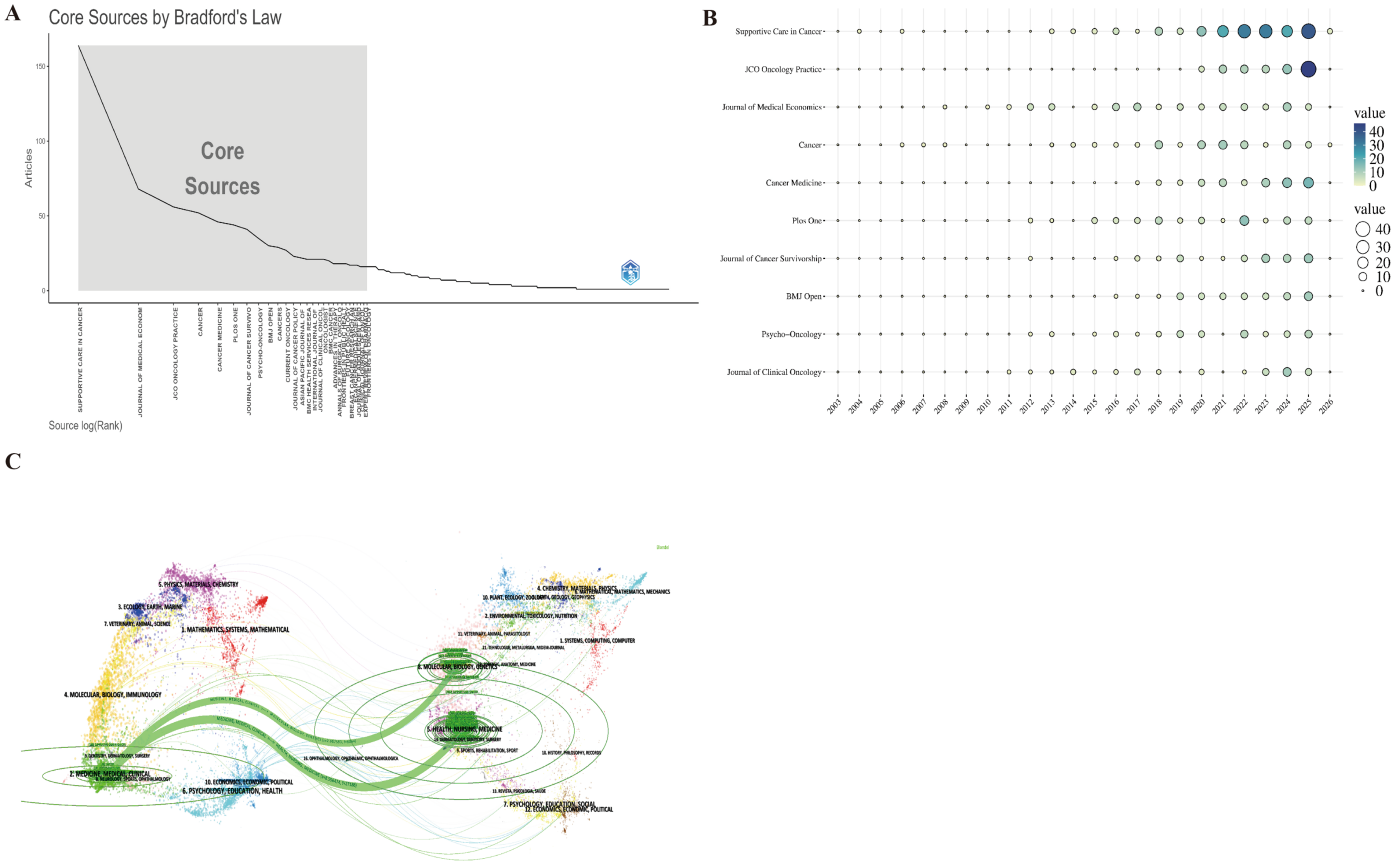
Journal distribution and citation landscape on financial toxicity in cancer patient research. **(A)** Core journals based on Bradford’s Law. **(B)** Publication trends from the top 10 most prolific academic journals. **(C)** Citation relationships across journal domains in financial toxicity research within cancer patient. On the left, clusters represent citing journal groups, while on the right, clusters represent the most frequently cited journals. Colored lines connecting the two maps illustrate the citation relationships between citing and cited journal clusters.

Journal analysis provides key insights into journal influence, publication trends, and research hotspots, guiding manuscript submission strategies and tracking disciplinary developments. Based on Bradford’s law (Figure 5A), the core journals in the field of FT among cancer patients were identified as Supportive Care in Cancer, Journal of Medical Economics, JCO Oncology Practice, Cancer, Cancer Medicine, PLOS One, Journal of Cancer Survivorship, Psycho-Oncology, BMJ Open, and Cancers. Annual publication output analysis among the top ten journals showed that Supportive Care in Cancer was the earliest to publish research on this topic (Figure 5B).

The dual-map overlay of journals (Figure 5C) illustrates the developmental trajectories and knowledge diffusion patterns in FT research for cancer patients. In this visualization, citing journals were primarily concentrated in the medicine, medical, and clinical fields, while cited journals were mainly distributed across molecular biology and genetics, as well as health, nursing, and medical sciences. The two green citation paths in the overlay represent the major directions of knowledge flow, indicating a gradual shift in research focus from traditional medicine and clinical disciplines to molecular biology, genetics, and health-related nursing and medical sciences.

### 3.5 Analysis of references

Reference analysis reveals a research field’s intellectual structure, thematic hotspots, interdisciplinary linkages, and academic influence, assisting researchers in understanding both its historical foundations and current state, while providing an evidence base for future research directions and strategic decision-making (24). To elucidate the knowledge base of FT research among cancer patients, the top 10 most highly cited articles in this field were systematically summarized (Table 1) (2, 6, 28–35). These influential studies cover the conceptual origins of FT, cancer-related cost quantification, population-level impacts, the development of measurement approaches, and the exploration of policy and intervention strategies. Early studies on the economic burden of cancer primarily focused on macroeconomic assessments, examining direct and indirect costs at the national or disease level, healthcare expenditures, and productivity losses. Seminal works by Luengo-Fernandez et al. and Yabroff et al. established the broader economic context in which patient-level financial hardship arises, providing an essential economic foundation for subsequent research (28, 34). A pivotal conceptual shift occurred with the pioneering work of Zafar et al. (2), who introduced a patient-centered framework for FT, defined its cost components, and linked financial burden to negative health outcomes, such as poor treatment adherence, diminished quality of life, increased psychological distress, and employment disruption. This marked a transition in oncology research, shifting focus from cost quantification to the real-world economic consequences of cancer treatment on patients. Concurrently, studies examining cost-sharing mechanisms and their downstream effects highlighted the importance of outcome-driven research (6, 30, 35). These highly cited studies reflect the evolution of FT research, from macroeconomic burden assessments to patient-centered financial distress, and advancing toward the development of measurement tools and evaluation strategies, firmly establishing FT as a multidimensional, clinically relevant consequence of cancer care.

**Table 1:** Knowledge structure of the top 10 highly cited studies on financial toxicity among cancer patients.

Rank	Article title	Source title	Author full names	Document type	DOI	Times Cited	Core focus
1	The Financial Toxicity of Cancer Treatment: A Pilot Study Assessing Out-of-Pocket Expenses and the Insured Cancer Patient’s Experience	Oncologist	Zafar, S. Yousuf; Peppercorn, Jeffrey M.; Schrag, Deborah; Taylor, Donald H.; Goetzinger, Amy M.; Zhong, Xiaoyin; Abernethy, Amy P.	Article	10.1634/theoncologist.2012-0279	1001	First to define financial toxicity as a treatment-related adverse effect and link it to patient quality of life and adherence
2	Economic burden of cancer across the European Union: a population-based cost analysis	Lancet Oncology	Luengo-Fernandez, Ramon; Leal, Jose; Gray, Alastair; Sullivan, Richard	Article	10.1016/S1470-2045(13)70442-X	835	Quantified direct, indirect, and productivity-related costs of cancer, providing macroeconomic context
3	The Financial Burden and Distress of Patients with Cancer: Understanding and Stepping-Up Action on the Financial Toxicity of Cancer Treatment	CA—A Cancer Journal for Clinicians	Carrera, Pricivel M.; Kantarjian, Hagop M.; Blinder, Victoria S.	Article	10.3322/caac.21443	784	Synthesized evidence on cost drivers, population impact, and policy implications of cancer-related financial burden
4	Measuring financial toxicity as a clinically relevant patient-reported outcome: The validation of the COmprehensive Score for financial Toxicity (COST)	Cancer	de Souza, Jonas A.; Yap, Bonnie J.; Wroblewski, Kristen; Blinder, Victoria; Araujo, Fabiana S.; Hlubocky, Fay J.; Nicholas, Lauren H.; O'Connor, Jeremy M.; Brockstein, Bruce; Ratain, Mark J.; Daugherty, Christopher K.; Cella, David	Article	10.1002/cncr.30369	750	Demonstrated prevalence and health consequences of financial toxicity across cancer populations
5	Financial Hardships Experienced by Cancer Survivors: A Systematic Review	JNCI—Journal of the National Cancer Institute	Altice, Cheryl K.; Banegas, Matthew P.; Tucker-Seeley, Reginald D.; Yabroff, K. Robin	Review	10.1093/jnci/djw205	730	Proposed pathways linking financial toxicity to treatment interruption and survival outcomes
6	Cost of care for older adult cancer patients in the United States	JNCI—Journal of the National Cancer Institute	Yabroff, K. Robin; Lamont, Elizabeth B.; Mariotto, Angela; Warren, Joan L.; Topor, Marie; Meekins, Angela; Brown, Martin L.	Article	10.1093/jnci/djn103	641	Examined treatment-related costs and insurance effects, informing early measurement approaches
7	The Development of a Financial Toxicity Patient-Reported Outcome in Cancer The COST Measure	Cancer	de Souza, Jonas A.; Yap, Bonnie J.; Hlubocky, Fay J.; Wroblewski, Kristen; Ratain, Mark J.; Cella, David; Daugherty, Christopher K.	Article	10.1002/cncr.28814	557	Linked economic hardship to quality of life and psychological outcomes
8	Burden of illness in cancer survivors: Findings from a population-based national sample	JNCI—Journal of the National Cancer Institute	Yabroff, KR; Lawrence, WF; Clauser, S; Davis, WW; Brown, ML	Article	10.1093/jnci/djh255	520	Demonstrated the impact of financial burden on treatment decisions and adherence
9	Financial Hardship Associated With Cancer in the United States: Findings From a Population-Based Sample of Adult Cancer Survivors	Journal of Clinical Oncology	Yabroff, K. Robin; Dowling, Emily C.; Guy, Gery P., Jr.; Banegas, Matthew P.; Davidoff, Amy; Han, Xuesong; Virgo, Katherine S.; McNeel, Timothy S.; Chawla, Neetu; Blanch-Hartigan, Danielle; Kent, Erin E.; Li, Chunyu; Rodriguez, Juan L.; de Moor, Janet S.; Zheng, Zhiyuan; Jemal, Ahmedin; Ekwueme, Donatus U.	Article	10.1200/JCO.2015.62.0468	460	Extended financial toxicity research to long-term cancer survivors
10	A Systematic Review of Financial Toxicity Among Cancer Survivors: We Can't Pay the Co-Pay	Patient—Patient Centered Outcomes Research	Gordon, Louisa G.; Merollini, Katharina M. D.; Lowe, Anthony; Chan, Raymond J.	Review	10.1007/s40271-016-0204-x	424	Evaluated potential interventions and health economic strategies to reduce financial toxicity

**Figure 6:**
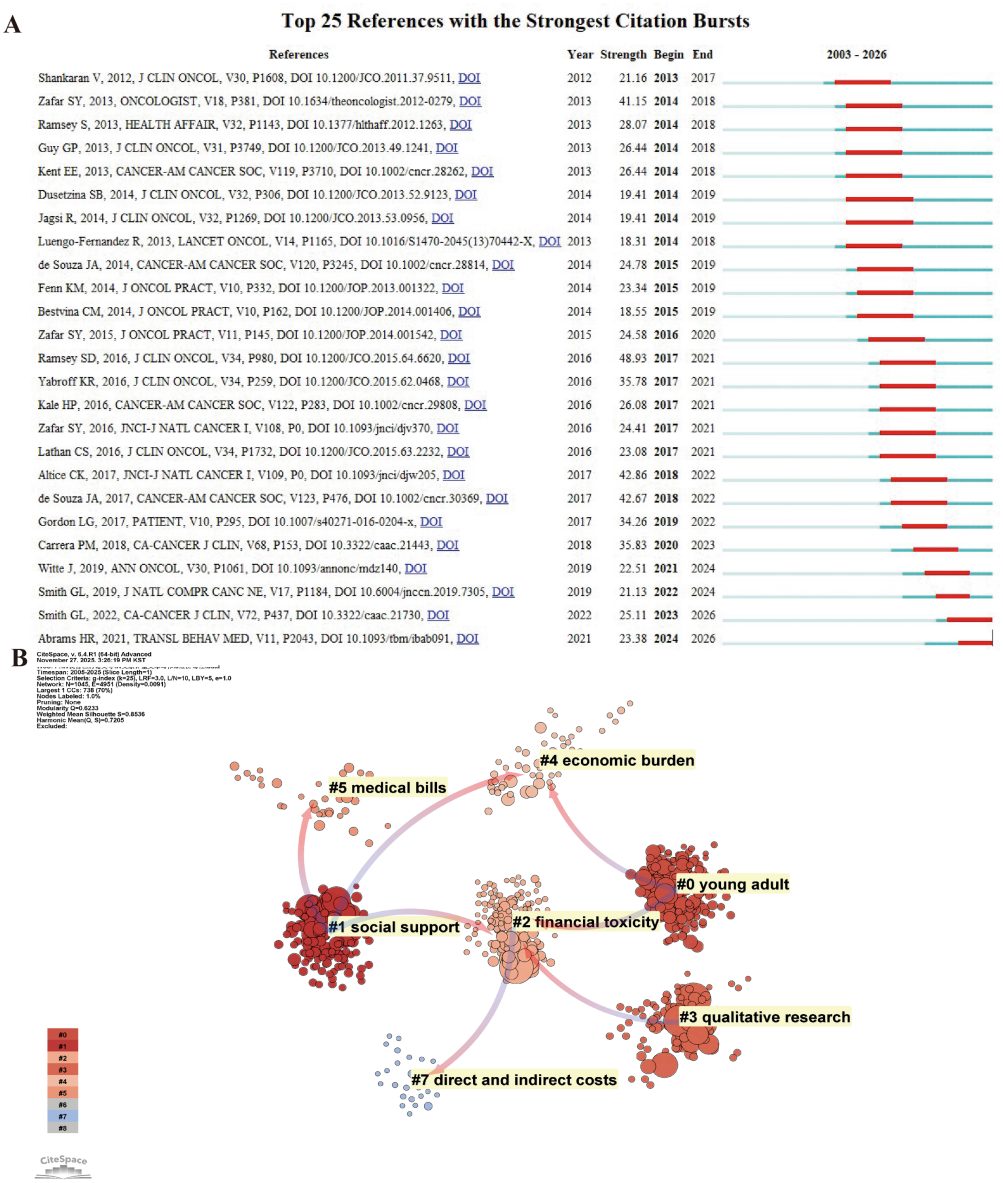
Intellectual structure and influential references in financial toxicity research. **(A)** Top 25 references with the strongest citation bursts from 2003 to 2026. **(B)** Reference clustering based on semantic similarity from 2003 to 2026, with pink arrows illustrating the primary flow of citations within each cluster.

Citation burst analysis further illustrates how research priorities have evolved over time. Among the Top 25 References with the Strongest Citation Bursts (Figure 6A), two articles demonstrated sustained citation bursts from 2023 to 2026 (11, 36), highlighting their central role in shaping current research directions. In Abrams et al. from Massachusetts General Hospital (36) systematically conceptualized FT as both a behavioral and structural determinant of cancer prognosis, clarifying its origins, mechanisms, and multilevel solutions. Their work identified key areas for future research, such as screening strategies, cost communication, integration into clinical workflows, and health equity–focused approaches, thus providing a theoretical and strategic framework for subsequent empirical studies, intervention research, and policy evaluation. Building on these conceptual advances, Smith et al. from MD Anderson Cancer Center (11) translated FT research into clinical practice by proposing a multidisciplinary management approach, framing FT as a modifiable risk factor in cancer care. This approach emphasizes patient-level risk assessments, financial navigation, and integrated care delivery strategies to mitigate FT in real-world settings. These burst references signal a paradigm shift in FT research, from early descriptive and measurement-focused studies to multidisciplinary, intervention-oriented frameworks, reflecting an increased focus on translating research into actionable management strategies within comprehensive cancer care.

Reference clustering analysis not only identifies current research hotspots but also visualizes their temporal evolution along a timeline, offering insights into the developmental trajectory of the field (Figure 6B). As illustrated in Figure 6B, Cluster #7 (direct and indirect costs) evolved into Cluster #2 (financial toxicity), which subsequently transformed into the rapidly expanding Cluster #1 (social support). A similar evolutionary path was observed from Cluster #4 (economic burden) to Cluster #0 (young adult), which is also undergoing rapid growth. These patterns highlight a paradigm shift in FT research, emphasizing a patient-centered and socially embedded perspective. Increasing attention is being given to social support mechanisms and vulnerable populations, particularly young adult cancer survivors, who now represent key emerging research hotspots.

### 3.6 Hotspots and trends

Keyword analysis plays a pivotal role in bibliometric research enabling the examination of the evolutionary trajectory developmental trends and emerging themes within a field (25, 37). The disciplinary analysis of keywords revealed pronounced interdisciplinary characteristics in FT research among cancer patients. Eight disciplines exhibited high betweenness centrality: oncology (0.59) public environmental and occupational health (0.53) molecular biology (0.19) pharmacology and pharmacy (0.14) biophysics (0.12) general and internal medicine (0.11) healthcare sciences and services (0.10) and nursing (0.10). These findings highlight extensive cross-disciplinary knowledge exchange and integration.

Keyword co-occurrence analysis identified high-frequency terms beyond the predefined search strategy such as care healthcare cost economic burden quality of life costs impact survivors health United States breast cancer mortality outcomes validation diagnosis prevalence women association cost of illness chemotherapy management therapy employment cost-effectiveness epidemiology out-of-pocket costs health insurance questionnaire psychology follow-up antineoplastic agents hospitalization healthcare utilization caregiver risk factors depression and social support. These findings suggest that current research is focused on specific cancer types and patient subgroups with significant emphasis on economic outcomes and patient-centered consequences. The strong connection between treatment intensity and financial burden highlights the growing recognition of the broader life impacts of cancer-related financial stress.

**Figure 7:**
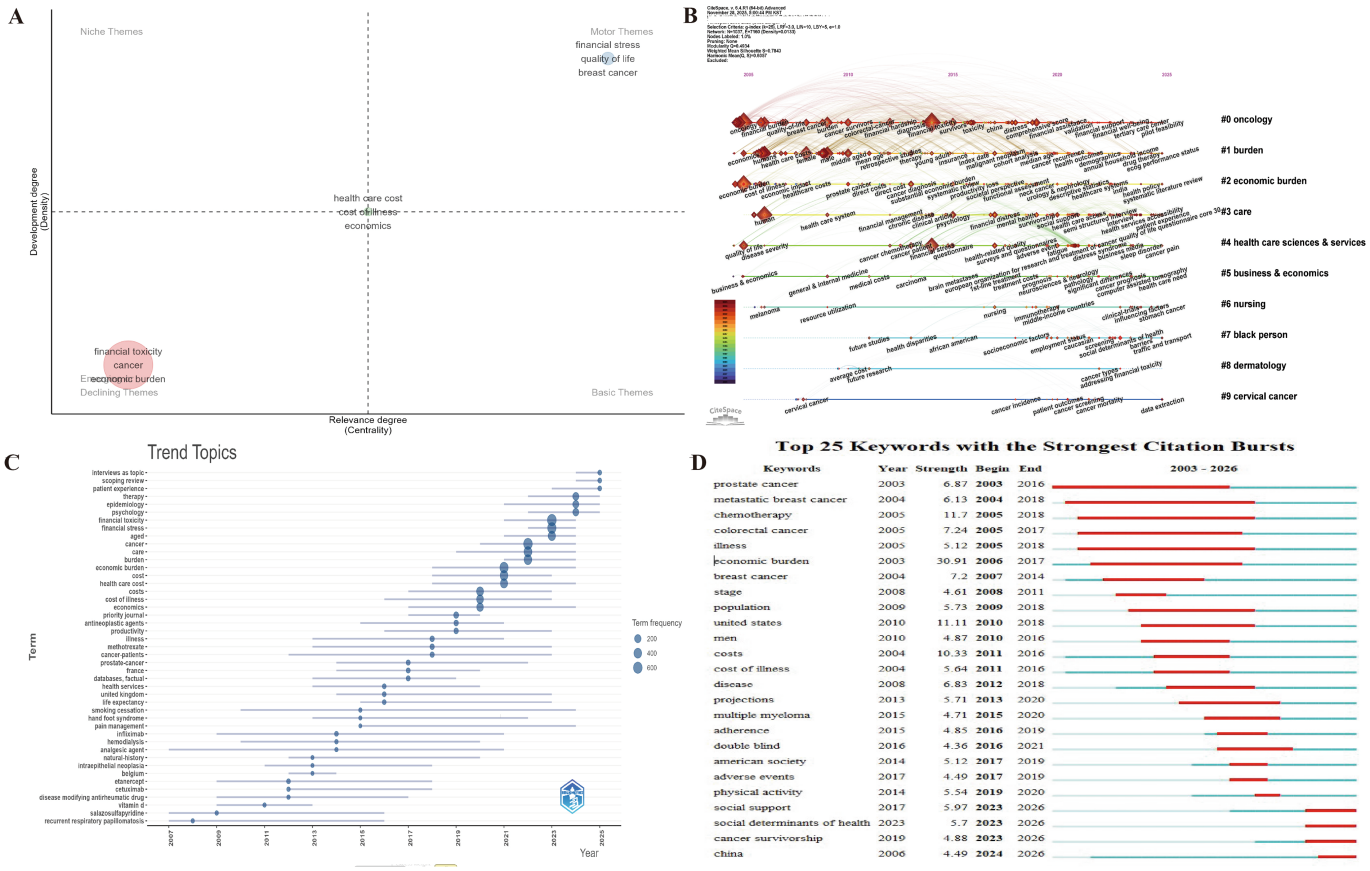
Thematic structure and evolution of research on financial toxicity in cancer. **(A)** Thematic map of research domains, illustrating the distribution of research themes based on centrality and density, identifying motor, basic, niche, and emerging or declining themes. **(B)** Timeline map of keyword clusters from 2003 to 2026. **(C)** Trend topics over time, depicting the temporal evolution of key research areas and highlighting emerging and declining areas of interest. **(D)** Top 25 keywords with the strongest citation bursts from 2003 to 2026.

Thematic map analysis further clarified the structural organization and developmental stages of key research themes in FT (Figure 7A). Financial stress, quality of life, and breast cancer emerged as motor themes, characterized by high centrality and density, indicating that these topics are both well-established and highly influential within the field. Conversely, FT and economic burden were identified as emerging themes, signaling that while these concepts are gaining attention, their thematic structures are still evolving. Topics such as healthcare cost, cost of illness, and economics occupied central positions on the thematic map, highlighting their foundational and cross-cutting roles across multiple research themes.

Keyword clustering analysis categorized keywords into ten major clusters (Figure 7B). The largest cluster #0 oncology included recent keywords such as pilot feasibility tertiary care center and financial support. Cluster #1 burden featured emerging terms like ECOG performance status drug therapy and annual household income. Cluster #2 economic burden highlighted recent keywords such as systematic literature review health policy and India while Cluster #3 care was associated with terms like quality of life questionnaire core 30 (EORTC QLQ-C30) patient experience and health services accessibility. These clusters can be further synthesized into four overarching research domains. First a prominent theme centers on the measurement and operationalization of financial toxicity reflected by keywords such as COST validation questionnaire and EORTC QLQ-C30. This cluster highlights sustained efforts to develop standardized instruments and adapt them across diverse healthcare systems and cultural contexts. Importantly this line of research underscores the ongoing challenge of achieving conceptual and measurement consistency in FT which remains a critical barrier to cross-study comparability and intervention evaluation. Second a major body of research focuses on the behavioral consequences of financial toxicity particularly cost-related medication nonadherence and treatment modification. Keywords such as treatment adherence income and patient experience indicate that FT directly influences patients’ clinical decision-making including delaying skipping or discontinuing recommended therapies. This theme provides crucial insight into the mechanisms through which FT translates into adverse clinical outcomes linking financial burden to survival and disease progression. Third a distinct and increasingly prominent theme involves the socioeconomic consequences of cancer-related financial burden including employment disruption income loss and long-term financial instability. Clusters associated with employment healthcare utilization social support and survivorship suggest that FT extends far beyond the treatment phase affecting patients’ ability to return to work maintain financial independence and sustain household stability. This highlights FT as not only a clinical issue but also a broader social and economic phenomenon. Fourth an emerging theme relates to intervention strategies and health system responses including financial navigation health policy and real-world evidence. Keywords such as pilot feasibility financial support and health policy indicate a shift toward designing and evaluating interventions aimed at mitigating FT. However the relative novelty and dispersion of these terms suggest that this area is still in an early developmental stage with limited high-quality evidence guiding best practices. Collectively these findings demonstrate that FT research has evolved from descriptive cost analyses to a more structured and mechanism-oriented field encompassing measurement development behavioral pathways socioeconomic consequences and intervention strategies. This thematic clarification provides a more precise understanding of how the field is organized and where future research efforts should be concentrated. Importantly these thematic clusters reveal that the field is converging on a clinically meaningful framework in which financial toxicity is understood not only as a measurable burden but as a determinant of treatment behavior and long-term socioeconomic outcomes.

Trend topic analysis revealed that during 2024–2025, prominent themes included interviews, scoping reviews, patient experience, therapy, epidemiology, and psychology (Figure 7C). Keyword burst detection in WoSCC identified social support, cancer survivorship, social determinants of health, and China as burst keywords from 2023 to 2026 (Figure 7D). Concurrently, VOSviewer burst analysis for the period 2022–2025 highlighted emerging terms such as real-world evidence, latent profile analysis, comprehensive score, and health-related social needs. These findings align closely with the reference burst analysis and identified hotspots in FT literature, reinforcing the robustness of the research frontiers. Collectively, they emphasize the growing influence of social support, cancer survivorship, and social determinants of health, alongside an increasing focus on advanced analytical methods, real-world data, and survivorship-oriented outcomes. Overall, keyword and hotspot analyses suggest that FT research has evolved toward a more interdisciplinary, patient-centered, and methodologically advanced direction. Current hotspots focus on psychosocial dimensions, survivorship, social determinants of health, and real-world evidence, while emerging analytical techniques and enhanced measurement tools are increasingly shaping future research trajectories. These insights provide a roadmap for future research priorities, particularly in developing targeted interventions and addressing unmet social and economic needs among cancer patients.

The temporal distribution of keyword bursts further highlights how research priorities in FT have shifted over time. Based on the time patterns of the Top 25 keywords with the strongest citation bursts, cancer-related FT research can be broadly divided into three distinct phases, each reflecting a transformation in research focus. In the earliest economic burden–oriented phase, keyword bursts centered on quantifying the economic impact of cancer and its treatment at the healthcare system or population level. Dominant terms included costs, disease, economic burden, cost of illness, healthcare, cost-effectiveness, chemotherapy, drugs, symptoms, models, prediction, countries, and United States. Research in this phase focused on treatment-related expenditures, cost-effectiveness analyses, and cross-national or healthcare system-level comparisons. Although the term “financial toxicity” had not yet been widely adopted, these studies provided foundational evidence of the significant economic consequences of cancer care. The subsequent patient-centered outcomes phase marked a shift toward patient-level and disease-specific analyses, with emerging terms such as cancer patients, breast cancer, prostate cancer, colorectal cancer, ovarian cancer, multiple myeloma, income, quality, and meta-analysis. This phase was characterized by increased attention to the heterogeneity of financial burden across different cancer types and patient populations. The most recent socio-structural survivorship phase of keyword bursts reflects a social and survivorship-oriented perspective, highlighted by terms such as social support, cancer survivorship, and social determinants of health. These keywords signal a shift toward examining the broader social context and long-term consequences of financial burden among cancer survivors. Research in this phase increasingly emphasizes how social and structural factors interact with financial stress across the cancer care continuum, particularly beyond the completion of active treatment. In summary, keyword burst analysis reveals a clear temporal evolution in research focus, from system-level cost assessments to patient-centered economic outcomes, and more recently to studies highlighting the social context and survivorship. However, these findings should be interpreted as reflecting publication patterns rather than causal relationships.

## 4 Discussion

### 4.1 Overall knowledge structure and evolution of research

In recent years, FT has emerged as a significant research focus in oncology, reflecting growing concerns about the economic impact of cancer and its treatment. This bibliometric analysis, based on data from the WoSCC and Scopus databases, offers a comprehensive overview of the knowledge structure, thematic evolution, and emerging research frontiers in FT among cancer patients. The findings indicate that FT research is a rapidly expanding field, marked by sustained scholarly attention. This growth parallels improvements in cancer survival, increasing treatment complexity, and rising healthcare expenditures, all of which have heightened awareness of the long-term financial implications of cancer care (38). Over time, FT has evolved from a secondary concern to a clinically relevant outcome that directly impacts treatment adherence, quality of life, and survival (39). Analyses at the national and institutional levels reveal significant geographical concentration, with high-income countries, particularly the USA and the UK, leading in publication output and collaboration networks. This dominance likely reflects the United States’ pioneering role in conceptualizing and empirically investigating FT, along with its earlier recognition of the challenges posed by escalating cancer care costs, gaps in insurance coverage, and household financial vulnerability, supported by well-established research infrastructures (2). Importantly, these findings also reflect underlying differences in healthcare systems and social safety nets: countries with broader insurance coverage and social welfare programs may experience and study FT differently from those where patients bear a higher proportion of out-of-pocket costs. For example, Cluster #2 prominently includes publications from India, where high out-of-pocket expenditures and limited social insurance coverage may amplify patient-reported financial distress and shape research priorities. Increasing participation from other regions signals growing global recognition of cancer-related financial hardship. Despite this, the limited extent of cross-regional collaboration highlights the need for broader and more inclusive international research partnerships that consider both economic contexts and health system structures.

Author collaboration analysis further highlights the central role of key contributors, particularly Professor K. Robin Yabroff and colleagues, whose work has been pivotal in advancing the understanding of cancer-related financial burden. Their research has focused on identifying modifiable factors at the patient, provider, health system, and policy levels that affect access to affordable cancer prevention, screening, treatment, survivorship care, and end-of-life services (40–42). Highly cited studies in this field are predominantly published in oncology, health services research, and interdisciplinary medical journals, reflecting the cross-cutting nature of FT research. The prominence of high-impact oncology and clinical journals further highlights the growing recognition of FT as a clinically relevant outcome, rather than merely an economic issue.

### 4.2 Hotspots and trends

Keyword co-occurrence and co-citation analyses revealed a coherent and progressive trajectory in the development of knowledge within this field. Early high-impact studies primarily focused on quantifying healthcare resource utilization out-of-pocket expenses and long-term costs thereby establishing economic burden as significant and pervasive aspect of cancer care. However over time the literature has increasingly shifted toward a patient-centered perspective emphasizing PROs social and structural determinants and actionable management strategies. This shift aligns closely with the rise of social support and multidisciplinary management as dominant research hotspots (11, 36). From a disciplinary standpoint FT is now widely regarded as a structural and equity-related issue within healthcare financing systems and broader social policy contexts rather than simply an individual financial burden (20). These trends suggest that FT should be understood not only as an economic risk but also as a determinant of patient experience and long-term well-being.

The convergence of social support-related themes with care-oriented and management-focused keywords highlights an ongoing shift from identifying risks to developing structured response strategies. Financial hardship often extends into the survivorship phase, interacting with factors such as employment status, insurance coverage, household resources, and access to supportive services. Consequently, multidisciplinary care models have become increasingly vital for alleviating financial distress (11, 14). In this context, social support is not merely a background variable but a modifiable factor within coordinated clinical workflows. Overall, these findings indicate that systematic social support assessment and multidisciplinary management have emerged as prominent research themes and promising conceptual directions within the FT literature. Future studies are warranted to further evaluate their potential roles in promoting patient-centered, equitable, and sustainable approaches to mitigating the economic burden of cancer care.

#### 4.2.1 Social support as a structural buffer against financial toxicity

As illustrated in Figures 6, 7, one of the most prominent and enduring foci identified in this study is social support, which is strongly linked to broader frameworks of social determinants of health. The sustained growth of co-citation clusters and keyword bursts related to social support reflects an increasing recognition that FT is deeply embedded within patients’ social and economic contexts. A growing body of research now conceptualizes FT as a socially patterned phenomenon, shaped by multiple structural factors, including household resources, employment security, insurance coverage, and access to supportive services (20, 36). Key empirical studies by Gordon et al. and Carrera et al. have been instrumental in advancing this perspective (29, 35). These studies consistently demonstrate that inadequate social support not only exacerbates financial strain during active treatment but also serves as a primary driver of ongoing financial hardship during the survivorship phase, particularly during post-treatment recovery. Consequently, financial recovery after cancer has been shown to be closely linked to social capital and patients’ ability to mobilize both formal and informal support systems, aligning with Khan et al.’s work on economic and social reintegration during cancer survivorship (14).

Recent conceptual advances have further integrated social support within broader social determinants of health frameworks, extending the theoretical interpretation of these empirical findings. One currently bursting reference, Abrams et al. (36), explicitly frames FT as a behavioral and structural determinant of cancer outcomes, positioning social support not just as a contextual variable but as a central target for intervention. This framework provides a vital conceptual anchor for understanding how social support interacts with healthcare systems, policy environments, and clinical practice to influence patients’ financial trajectories. The concurrent emergence of keywords such as social support, health policy, and healthcare accessibility further reinforces this structural interpretation. This trend is closely aligned with recent shifts in oncology practice, including routine screening of patients’ social needs, integration of community and social resources into cancer care pathways, and increased focus on addressing structural vulnerabilities among socioeconomically disadvantaged populations within supportive care models (43, 44). These findings indicate that social support has evolved from a descriptive correlate of FT to a central conceptual and practical pillar with clear intervention relevance. Future research is likely to increasingly focus on the design and evaluation of structured social support strategies, including financial navigation programs, system-level efforts to enhance access to supportive services, and policy-level interventions targeting social inequities. Translating theoretical and empirical insights on social support into sustainable and scalable care models will be essential for improving the financial well-being and long-term outcomes of cancer patients on a broader scale.

#### 4.2.2 Multidisciplinary management and clinical integration of financial toxicity in patients with cancer

Building on the structural understanding of social support as a determinant of financial vulnerability, another major research hotspot identified in this bibliometric analysis is multidisciplinary management as an effective approach to addressing FT. This shift reflects a broader transition in the field from merely identifying and describing financial hardship to developing organized, sustainable mitigation strategies integrated within routine cancer care. Among the most highly cited publications, Carrera et al. (29) redirected attention to health system–level responsibility and emphasized the need for cross-disciplinary collaboration in addressing FT. This conceptual evolution has been translated into actionable clinical pathways by one of the currently highly cited references, Smith et al. (11), who proposed a multidisciplinary management framework that frames FT as an identifiable, stratifiable, and modifiable clinical risk factor within oncology workflows. This perspective aligns with initiatives advocated by the American Society of Clinical Oncology (ASCO) and the broader field of supportive oncology, which increasingly stress the integration of economic considerations into cancer treatment decision-making and longitudinal follow-up (8, 20). These developments highlight the importance of multidisciplinary management as a structural response to FT. Thematic and keyword analyses further reveal a growing synergy between multidisciplinary management and patient-centered outcome assessment. The prominence of quality of life as a central theme, alongside emerging keywords such as patient experience and EORTC QLQ-C30, indicates that FT is increasingly being assessed through validated PRO measures. Concurrent methodological advances, including real-world evidence, latent profile analysis, and comprehensive scoring systems, signal a shift toward risk stratification and personalized intervention pathways.

Notably, these trends toward risk stratification and personalized intervention pathways parallel the broader evolution of precision and personalized oncology. Advances in targeted therapies and immunotherapies have substantially improved clinical outcomes but are often associated with significantly higher and more variable costs (45, 46). As a result, financial toxicity is increasingly intertwined with treatment decision-making, particularly in contexts where multiple therapeutic options with differing cost profiles are available. In this regard, the integration of financial considerations into precision oncology gives rise to the concept of “precision financial toxicity management,” in which economic risk is assessed alongside clinical and biological factors. This approach emphasizes the need for early financial risk stratification, incorporation of cost discussions into shared decision-making, and the use of PROs to dynamically monitor financial distress across the treatment trajectory. By aligning personalized treatment strategies with individualized financial assessment and support, clinicians can better balance therapeutic efficacy with affordability and patient preferences. Such integration is particularly relevant in the era of value-based cancer care, where optimizing both clinical outcomes and economic sustainability is essential. Embedding financial navigation, insurance counseling, and cost-transparency tools into precision oncology workflows may represent a critical step toward reducing disparities in access to innovative therapies and improving overall patient-centered outcomes.

Notably, the rapid growth of the young adult reference cluster highlights the particular relevance of multidisciplinary approaches for this population, as younger cancer survivors often face unique challenges related to employment disruption, insurance instability, and long-term financial consequences (47, 48). However, the implementation of multidisciplinary management strategies remains heterogeneous across countries and even among institutions within the same healthcare system (49). This variability highlights the need for greater coordination and standardization of diagnostic and treatment pathways to effectively manage FT.

Overall, multidisciplinary management is evolving from a conceptual advocacy framework into a practice-oriented model that aligns closely with contemporary goals of supportive oncology and value-based cancer care. The two major research hotspots identified in this bibliometric analysis, social support and multidisciplinary management represent complementary dimensions of a unified conceptual framework for FT research and practice. Social support constitutes the upstream structural context shaping baseline financial vulnerability, while multidisciplinary management operationalizes this understanding through coordinated clinical and system-level interventions. Effective mitigation of FT requires early identification of social and economic risk factors, seamless integration of financial assessment into clinical workflows, and coordinated interventions across clinical, administrative, and policy domains. By bridging social determinants with clinical management, FT is increasingly positioned as a legitimate and actionable target within comprehensive cancer care. This paradigm reflects a broader alignment with patient-centered, value-based oncology models that prioritize quality of life, survivorship outcomes, and health equity alongside traditional clinical endpoints.

### 4.3 Conceptual model: a framework for managing financial toxicity in patients with cancer

Drawing on the integrated evidence from this bibliometric analysis, a conceptual model is proposed that frames FT as a dynamic and modifiable risk influenced by interactions across social, clinical, and health system levels. The three-phase progression observed in keyword bursts from system-level cost analysis to patient-centered outcomes and, more recently, to social and survivorship contexts provides the empirical basis for this model. Within this framework, FT can be understood as a dynamic and modifiable condition shaped by multi-level factors. At the upstream level, social determinants such as socioeconomic status, employment stability, insurance coverage, and social support networks, may shape patients’ baseline financial vulnerability at the time of cancer diagnosis. At the clinical level, FT has been increasingly associated in the literature with treatment-related costs, healthcare utilization patterns, and disruptions to work capacity and daily functioning. The growing use of patient-reported outcome measures in FT research further suggests increasing scholarly interest in the early identification and monitoring of financial distress within oncology care. The recent shift toward survivorship and social-context-oriented themes also highlights increasing attention to the long-term and structural dimensions of FT across the cancer care continuum. In this context, multidisciplinary management and social support have emerged as prominent conceptual directions in the literature, involving collaboration among oncologists, nurses, social workers, financial navigators, and administrative personnel. These approaches have been increasingly discussed as potential strategies for addressing the complex and multidimensional nature of FT. In addition, real-world data and patient-reported outcomes may provide valuable insights for refining FT-related assessment and management approaches in future research and clinical practice. Importantly, the proposed framework should be interpreted as hypothesis-generating rather than explanatory or causal. It is intended to synthesize the dominant thematic patterns and conceptual relationships identified through bibliometric analysis and to provide a structured foundation for future empirical validation, intervention studies, and policy-oriented research.

### 4.4 Implications for future research and practice

This bibliometric study, by mapping the knowledge evolution and conceptual integration of FT research, offers crucial insights for future empirical, clinical, and policy-oriented endeavors. The findings emphasize the need for rigorous evaluation of multidisciplinary interventions, the standardization of social and financial risk screening within oncology care, and the expanded use of real-world data to guide personalized management strategies. These directions reflect a growing consensus that FT is a preventable and manageable aspect of comprehensive, equity-driven oncology practice. Clinically, the results highlight the importance of integrating routine financial risk screening, structured financial navigation, and social needs assessments into standardized oncology care pathways. Incorporating these components into clinical workflows can ensure consistent identification and proactive management of FT, enhancing patient-centered care and promoting equity in cancer outcomes. From a system perspective, these findings suggest opportunities to align supportive oncology services with broader value-based care initiatives that prioritize long-term survivorship and quality of life alongside traditional clinical outcomes.

Importantly, beyond these structural and system-level implications, it is essential to recognize the human dimension of financial toxicity, which is often not fully captured by bibliometric or quantitative analyses. For many patients and their families, FT is embedded in daily life and manifests through difficult trade-offs between cancer treatment and essential life commitments, such as housing, employment, transportation, and family responsibilities. These lived experiences involve emotional, social, and ethical considerations that extend beyond measurable financial indicators. The potential disconnect between high-level research outputs and individual patient experiences underscores the need for future research to incorporate qualitative and mixed-methods approaches, including patient narratives and longitudinal assessments of financial coping. Integrating these perspectives will help ensure that FT-related interventions and policies are not only evidence-based but also grounded in the realities of patients’ daily lives.

Despite the growing volume of research, several critical gaps remain. In particular, intervention-based studies addressing FT are still limited, and there is a lack of standardized measurement frameworks across different healthcare systems. In addition, low- and middle-income countries remain underrepresented in the literature, potentially limiting the global generalizability of current evidence. Addressing these gaps will be essential for advancing FT research from descriptive characterization toward actionable and equity-oriented solutions.

### 4.5 Limitations

This study provides a thorough overview of the knowledge structure, thematic evolution, and emerging research frontiers on cancer-related FT, utilizing the WoSCC and Scopus databases. However, several limitations must be acknowledged. First, bibliometric analyses are inherently constrained by the quality and scope of available metadata, which may not fully capture the theoretical or methodological subtleties of individual studies. Second, citation-based indicators may disproportionately favor earlier publications or studies from high-income countries, potentially underrepresenting emerging contributions from low- and middle-income nations—regions that often bear a disproportionate burden of FT.

## 5 Conclusion

In conclusion, this bibliometric study systematically analyzed the literature on FT among cancer patients, identifying the most influential countries, institutions, authors, and journals in the field. It characterized international collaboration patterns, outlined the research domain’s evolutionary trajectory, and highlighted key research hotspots and emerging frontiers. The findings reveal a sharp increase in annual publications in recent years, emphasizing the growing significance of the topic and its continued prominence in academic discourse. Notably, the field has evolved conceptually, shifting from an initial focus on economic burden and cost quantification to a more patient-centered, system-oriented understanding of FT as a modifiable risk factor within social and clinical contexts. Social support and multidisciplinary management were identified as two interconnected research hotspots, reflecting the increasing recognition that mitigating FT effectively requires both an upstream focus on social determinants of health and downstream integration of coordinated clinical interventions. The proposed conceptual model further clarifies how social vulnerability, clinical assessment, and multidisciplinary management can be integrated to improve quality of life, patient experience, and survivorship outcomes.

These findings deepen the theoretical understanding of FT and provide a coherent conceptual foundation for future empirical research, clinical application, and health system improvement. As the global cancer survivor population grows, addressing FT through integrated, patient-centered, and multidisciplinary approaches will be critical for advancing equitable and high-quality cancer care. This study offers an objective, comprehensive perspective on the FT research landscape and provides valuable insights for informing policy development, resource allocation, and continued advancements in the field.

## Data Availability

The original contributions presented in the study are included in the article/supplementary material, further inquiries can be directed to the corresponding author.

## References

[1] Zafar SY Abernethy AP. Financial toxicity, part I: a new name for a growing problem. Oncology (Williston Park). (2013) 27:80–149. 23530397 PMC4523887

[2] Zafar SY Peppercorn JM Schrag D Taylor DH Goetzinger AM Zhong X . The financial toxicity of cancer treatment: a pilot study assessing out-of-pocket expenses and the insured cancer patient's experience. Oncologist. (2013) 18:381–90. doi: 10.1634/theoncologist.2012-0279, 23442307 PMC3639525

[3] Wagle NS Nogueira L Devasia TP Mariotto AB Yabroff KR Islami F . Cancer treatment and survivorship statistics, 2025. CA Cancer J Clin. (2025) 75:308–40. doi: 10.3322/caac.70011, 40445120 PMC12223361

[4] Ehsan AN Wu CA Minasian A Singh T Bass M Pace L . Financial toxicity among patients with breast cancer worldwide: a systematic review and meta-analysis. JAMA Netw Open. (2023) 6:e2255388. doi: 10.1001/jamanetworkopen.2022.55388, 36753274 PMC9909501

[5] Mariotto AB Enewold L Zhao J Zeruto CA Yabroff KR. Medical care costs associated with cancer survivorship in the United States. Cancer Epidemiol Biomarkers Prev. (2020) 29:1304–12. doi: 10.1158/1055-9965.EPI-19-1534, 32522832 PMC9514601

[6] Altice CK Banegas MP Tucker-Seeley RD Yabroff KR. Financial hardships experienced by cancer survivors: a systematic review. J Natl Cancer Inst. (2017) 109:djw205. doi: 10.1093/jnci/djw205, 27754926 PMC6075571

[7] Witte J Mehlis K Surmann B Lingnau R Damm O Greiner W . Methods for measuring financial toxicity after cancer diagnosis and treatment: a systematic review and its implications. Ann Oncol. (2019) 30:1061–70. doi: 10.1093/annonc/mdz140, 31046080 PMC6637374

[8] Wagstaff A Neelsen S. A comprehensive assessment of universal health coverage in 111 countries: a retrospective observational study. Lancet Glob Health. (2020) 8:e39–49. doi: 10.1016/S2214-109X(19)30463-2, 31837954

[9] Yabroff KR Mariotto A Tangka F Zhao J Islami F Sung H . Annual report to the nation on the status of cancer, part 2: patient economic burden associated with cancer care. J Natl Cancer Inst. (2021) 113:1670–82. doi: 10.1093/jnci/djab192, 34698839 PMC9891103

[10] Noronha V Tongaonkar A Pillai A Rao AR Kumar A Sehgal A . Prevalence and impact of financial toxicity in older patients with cancer: a prospective observational study in India. Support Care Cancer. (2025) 33:416. doi: 10.1007/s00520-025-09252-9, 40278900 PMC12031899

[11] Smith GL Banegas MP Acquati C Chang S Chino F Conti RM . Navigating financial toxicity in patients with cancer: a multidisciplinary management approach. CA Cancer J Clin. (2022) 72:437–53. doi: 10.3322/caac.21730, 35584404 PMC12994614

[12] Desai A Gyawali B. Financial toxicity of cancer treatment: moving the discussion from acknowledgement of the problem to identifying solutions. EClinicalMedicine. (2020) 20:100269. doi: 10.1016/j.eclinm.2020.100269, 32300733 PMC7152810

[13] Voros K Skubic M Bavdaz M Dosenovic Bonca P Perhavec A Redek T . Assessment of financial toxicity in patients with cancer in Slovenia. Support Care Cancer. (2025) 33:515. doi: 10.1007/s00520-025-09591-7, 40445419 PMC12125031

[14] Khan HM Ramsey S Shankaran V. Financial toxicity in cancer care: implications for clinical care and potential practice solutions. J Clin Oncol. (2023) 41:3051–8. doi: 10.1200/JCO.22.01799, 37071839

[15] Scilipoti P Moschini M Li R Lerner SP Black PC Necchi A . The financial burden of localized and metastatic bladder cancer. Eur Urol. (2025) 87:536–50. doi: 10.1016/j.eururo.2024.12.002 39730299

[16] Uppal N Collins R James B. Thyroid nodules: global, economic, and personal burdens. Front Endocrinol (Lausanne). (2023) 14:1113977. doi: 10.3389/fendo.2023.1113977, 36755911 PMC9899850

[17] Patel KB Turner K Alishahi Tabriz A Gonzalez BD Oswald LB Nguyen OT . Estimated indirect cost savings of using telehealth among nonelderly patients with cancer. JAMA Netw Open. (2023) 6:e2250211. doi: 10.1001/jamanetworkopen.2022.50211, 36626174 PMC9856804

[18] Carrera PM Curigliano G Santini D Sharp L Chan RJ Pisu M . ESMO expert consensus statements on the screening and management of financial toxicity in patients with cancer. ESMO Open. (2024) 9:102992. doi: 10.1016/j.esmoop.2024.102992, 38626634 PMC11033153

[19] Narasimman M Hernaez R Cerda V Lee M Yekkaluri S Khan A . Financial burden of hepatocellular carcinoma screening in patients with cirrhosis. Clin Gastroenterol Hepatol. (2024) 22:760–767.e1. doi: 10.1016/j.cgh.2023.07.018, 37544418 PMC12206404

[20] Klimeck L Heisser T Hoffmeister M Brenner H. Colorectal cancer: a health and economic problem. Best Pract Res Clin Gastroenterol. (2023) 66:101839. doi: 10.1016/j.bpg.2023.101839, 37852707

[21] Donthu N Kumar S Mukherjee D Pandey N Lim WM. How to conduct a bibliometric analysis: an overview and guidelines. J Bus Res. (2021) 133:285–96. doi: 10.1016/j.jbusres.2021.04.070

[22] Cheng H Lin L Liu T Wang S Zhang Y Tian L. Financial toxicity of breast cancer over the last 30 years: a bibliometrics study and visualization analysis via CiteSpace. Medicine (Baltimore). (2023) 102:e33239. doi: 10.1097/MD.0000000000033239, 36961181 PMC10036026

[23] Ratosa I Bavdaz M Bonca PD Logar HBZ Perhavec A Skubic M . The financial toxicity of breast cancer: a systematic mapping of the literature and identification of research challenges. Radiol Oncol. (2025) 59:31–42. doi: 10.2478/raon-2025-0002, 39754600 PMC11867575

[24] Chen C Ibekwe-SanJuan F Hou J. The structure and dynamics of cocitation clusters: a multiple-perspective cocitation analysis. J Am Soc Inf Sci Technol. (2010) 61:1386–409. doi: 10.1002/asi.21309

[25] Chen C Song M. Visualizing a field of research: a methodology of systematic scientometric reviews. PLoS One. (2019) 14:e0223994. doi: 10.1371/journal.pone.0223994, 31671124 PMC6822756

[26] Chen CM. CiteSpace II: detecting and visualizing emerging trends and transient patterns in scientific literature. J Am Soc Inf Sci Technol. (2006) 57:359–77. doi: 10.1002/asi.20317

[27] van Eck NJ Waltman L. Software survey: VOSviewer, a computer program for bibliometric mapping. Scientometrics. (2010) 84:523–38. doi: 10.1007/s11192-009-0146-3, 20585380 PMC2883932

[28] Luengo-Fernandez R Leal J Gray A Sullivan R. Economic burden of cancer across the European Union: a population-based cost analysis. Lancet Oncol. (2013) 14:1165–74. doi: 10.1016/S1470-2045(13)70442-X, 24131614

[29] Carrera PM Kantarjian HM Blinder VS. The financial burden and distress of patients with cancer: understanding and stepping-up action on the financial toxicity of cancer treatment. CA Cancer J Clin. (2018) 68:153–65. doi: 10.3322/caac.21443, 29338071 PMC6652174

[30] de Souza JA Yap BJ Wroblewski K Blinder V Araujo FS Hlubocky FJ . Measuring financial toxicity as a clinically relevant patient-reported outcome: the validation of the comprehensive score for financial toxicity (cost). Cancer. (2017) 123:476–84. doi: 10.1002/cncr.30369, 27716900 PMC5298039

[31] Yabroff KR Lamont EB Mariotto A Warren JL Topor M Meekins A . Cost of care for elderly cancer patients in the United States. J Natl Cancer Inst. (2008) 100:630–41. doi: 10.1093/jnci/djn103, 18445825

[32] de Souza JA Yap BJ Hlubocky FJ Wroblewski K Ratain MJ Cella D . The development of a financial toxicity patient-reported outcome in cancer: the cost measure. Cancer. (2014) 120:3245–53. doi: 10.1002/cncr.28814, 24954526

[33] Yabroff KR Lawrence WF Clauser S Davis WW Brown ML. Burden of illness in cancer survivors: findings from a population-based national sample. J Natl Cancer Inst. (2004) 96:1322–30. doi: 10.1093/jnci/djh255, 15339970

[34] Yabroff KR Dowling EC Guy GP Jr Banegas MP Davidoff A Han X . Financial hardship associated with cancer in the United States: findings from a population-based sample of adult cancer survivors. J Clin Oncol. (2016) 34:259–67. doi: 10.1200/JCO.2015.62.0468, 26644532 PMC4872019

[35] Gordon LG Merollini KMD Lowe A Chan RJ. A systematic review of financial toxicity among cancer survivors: we can't pay the co-pay. Patient. (2017) 10:295–309. doi: 10.1007/s40271-016-0204-x, 27798816

[36] Abrams HR Durbin S Huang CX Johnson SF Nayak RK Zahner GJ . Financial toxicity in cancer care: origins, impact, and solutions. Transl Behav Med. (2021) 11:2043–54. doi: 10.1093/tbm/ibab091, 34850932

[37] Ellegaard O Wallin JA. The bibliometric analysis of scholarly production: how great is the impact? Scientometrics. (2015) 105:1809–31. doi: 10.1007/s11192-015-1645-z, 26594073 PMC4643120

[38] Diao X Guo C Jin Y Li B Gao X Du X . Cancer situation in China: an analysis based on the global epidemiological data released in 2024. Cancer Commun. (2025) 45:178–97. doi: 10.1002/cac2.12627, 39659114 PMC11833671

[39] Sherman RL Firth AU Henley SJ Siegel RL Negoita S Sung H . Annual report to the nation on the status of cancer, featuring state-level statistics after the onset of the COVID-19 pandemic. Cancer. (2025) 131:e35833. doi: 10.1002/cncr.35833, 40257373 PMC12010951

[40] Lu AD Zheng Z Han X Qi R Zhao J Yabroff KR . Medical financial hardship in survivors of adolescent and young adult cancer in the United States. J Natl Cancer Inst. (2021) 113:997–1004. doi: 10.1093/jnci/djab013, 33839786 PMC8328985

[41] Yabroff KR Han X Song W Zhao J Nogueira L Pollack CE . Association of Medical Financial Hardship and Mortality among cancer survivors in the United States. J Natl Cancer Inst. (2022) 114:863–70. doi: 10.1093/jnci/djac044, 35442439 PMC9194618

[42] Graboyes EM Chaiyachati KH Sisto Gall J Johnson W Krishnan JA McManus SS . Addressing transportation insecurity among patients with cancer. J Natl Cancer Inst. (2022) 114:1593–600. doi: 10.1093/jnci/djac134, 36130286 PMC9745432

[43] Marshall VK Tofthagen C Advani P Mussallem D Zambroski C Visovsky C. Qualitative evaluation of financial toxicity and supportive care needs of women living with metastatic breast Cancer. Nurs Res. (2025) 74:266–71. doi: 10.1097/NNR.0000000000000831, 40307226

[44] Ilkhani S Naus AE Pinkes N Rafaqat W Grobman B Valverde MD . The invisible scars: unseen financial complications worsen every aspect of long-term health in trauma survivors. J Trauma Acute Care Surg. (2024) 96:893–900. doi: 10.1097/TA.0000000000004247, 38227675

[45] Silveira A Sequeira T Goncalves J Lopes Ferreira P. Patient reported outcomes in oncology: changing perspectives-a systematic review. Health Qual Life Outcomes. (2022) 20:82. doi: 10.1186/s12955-022-01987-x, 35597948 PMC9124403

[46] Puccini A Viscardi G Ciani O Efficace F Piattelli A Beretta GD . Patient-reported outcomes (Pros) in clinical trials and in clinical practice: report from the xxi National Conference of the Italian Association of Medical Oncology (Aiom). BMJ Oncol. (2025) 4:e000783. doi: 10.1136/bmjonc-2025-000783, 40546927 PMC12182000

[47] Eng C Jacome AA Agarwal R Hayat MH Byndloss MX Holowatyj AN . A comprehensive framework for early-onset colorectal cancer research. Lancet Oncol. (2022) 23:35090673:e116–28. doi: 10.1016/S1470-2045(21)00588-X 35090673

[48] Hughes K Ford K Bellis MA Glendinning F Harrison E Passmore J. Health and financial costs of adverse childhood experiences in 28 European countries: a systematic review and Meta-analysis. Lancet Public Health. (2021) 6:e848–57. doi: 10.1016/S2468-2667(21)00232-2, 34756168 PMC8573710

[49] Rimassa L Khan S Groot Koerkamp B Roessler S Andersen JB Raggi C . Mapping the landscape of biliary tract cancer in Europe: challenges and controversies. Lancet Reg Health Eur. (2025) 50:101171. doi: 10.1016/j.lanepe.2024.101171, 40093398 PMC11910794

